# Intercropping tea plants with different leguminous green manures enhances soil nutrient availability, thereby reshaping the structure and functional potential of soil microbial communities

**DOI:** 10.3389/fmicb.2026.1700016

**Published:** 2026-02-10

**Authors:** Qin Liu, Li-xian Wang, Pei-yu Chang, Jian-gen Zhang, Chen Li, Qiao-yun Shuang, Chun-yun Zhang, Zhi-Kai Lu, Xiao-ling Wang, Xin-feng Jiang

**Affiliations:** 1 Jiangxi Provincial Key Laboratory of Plantation and High Valued Utilization of Specialty Fruit Tree and Tea, Cash Crops Research Institute of Jiangxi Province, Nanchang, China; 2 Research Center for Ecological Science, Jiangxi Agricultural University, Nanchang, China; 3 Institute of Biological Resources, Jiangxi Academy of Sciences, Nanchang, China

**Keywords:** enzyme activity, intercropping, leguminous green manure, microbial community, soil fertility, tea plant

## Abstract

Long-term monoculture in tea plantations leads to soil fertility decline and microbial community degradation, restricting tea growth and ecosystem function. To explore sustainable management strategies, we conducted a field experiment in a 40-year-old *Camellia sinensis* cv. “Fuding Dabai” plantation with intercropping of three leguminous green manures: alfalfa (TAL), hairy vetch (THV), and Chinese milk vetch (TMV). All treatments significantly improved bud density, hundred-bud weight, and yield, with TAL and THV increasing spring and summer yields by more than 40% and THV achieving the highest autumn yield (650.17 kg·hm^−2^). Soil fertility was markedly enhanced, particularly under THV, with notable increases in organic matter, nitrogen, and available phosphorus. Enzyme activities responded differentially: TAL enhanced urease and sucrase, TMV promoted amylase and phosphatase, and THV increased catalase activity. High-throughput sequencing revealed shifts in bacterial (*Pseudomonadota*, *Planctomycetota*) and fungal (*Ascomycota*, *Basidiomycota*) communities, with THV showing the highest bacterial OTU richness. Functional predictions indicated enhanced microbial potential in carbon, nitrogen, and phosphorus cycling, especially under THV. Overall, leguminous green manure intercropping improved soil fertility, enzyme activity, and microbial communities, thereby enhancing tea yield, with hairy vetch showing the most consistent benefits. These findings provide insights into microbial-mediated soil improvement and offer a sustainable pathway for managing aging tea plantations.

## 1 Introduction

Tea is one of the most widely consumed beverages worldwide, and the sustainable development of the tea industry largely depends on maintaining soil ecosystem functions. As a perennial cash crop system, the long-term stable production of tea plantations is closely linked to soil fertility and ecological functionality. However, long-term monoculture is widespread in tea plantations, and such management practices often lead to soil acidification, nutrient imbalance, and reduced microbial diversity, thereby diminishing nutrient availability and ecosystem stability (Gui et al., 2022; Jibola-Shittu et al., 2024; Zhao et al., 2018). This acidification is driven in part by intensive synthetic nitrogen fertilizer use, which releases hydrogen ions during ammonium nitrification, and by tea plant root exudates that contain organic acids. Moreover, continuous nutrient uptake by tea monocultures without sufficient organic matter replenishment weakens the soil’s buffering capacity, further exacerbating acidification (Wang et al., 2025a, 2025b, 2025c). Substantial evidence has shown that continuous monoculture in cropping systems, such as maize, coffee, or tomato, can exacerbate soil acidification. In addition, it can significantly alter the structure of microbial communities (Behnke et al., 2020; Fu et al., 2017; Yang et al., 2025). Moreover, soil acidification not only suppresses the functions of beneficial microbial communities but also reduces crop yield and quality, making it a key constraint to the sustainable development of tea plantations (Huang et al., 2023a, 2023b; Zhang et al., 2022, 2023).

Among sustainable agricultural practices, leguminous green manures are considered an important approach to improving soil quality due to their functions in biological nitrogen fixation, nutrient cycling, and organic matter accumulation (Yuan et al., 2023). Previous studies have demonstrated that green manure intercropping can effectively increase soil organic carbon and total nitrogen contents, enhance the activity of key enzymes involved in C, N, and P cycling such as urease and phosphatase, and promote microbial community diversity (Shao et al., 2024; Wang et al., 2025a, 2025b, 2025c). In recent years, the role of leguminous green manures in tea plantation systems has attracted increasing attention. Wang T. et al. (2023) reported that intercropping legumes in tea plantations could improve soil physicochemical properties and enhance tea quality by regulating rhizosphere microbial communities. Zhou et al. (2024) further demonstrated that green manure intercropping enhanced soil enzyme activity and bacterial diversity while improving quality-related metabolites such as amino acids and tea polyphenols. Another study showed that intercropping leguminous green manures significantly increased soil microbial diversity and enhanced amino acid and soluble sugar contents in tea leaves (Wang T. et al., 2023; Wang S. et al., 2023). Such metabolite changes are often mediated by shifts in soil microbial activity and nutrient availability, further supporting the ecological mechanisms examined in this study. In degraded tea plantations, combining green manure with organic amendments also showed strong restorative potential. For instance, Jiang et al. (2019) revealed that co-application of green manure and goat manure increased microbial biomass and enzyme activity, promoted organic acid secretion, and improved soil fertility. Similarly, Shao et al. (2024) indicated that green manure cover promoted soil organic matter accumulation and carbon sequestration.

Overall, intercropping legumes with tea not only improves soil physicochemical properties and fertility but also enhances enzyme activity, optimizes microbial community structure, and indirectly improves tea quality (Chen et al., 2025; Zhou et al., 2024; Wang et al., 2025a, 2025b, 2025c). However, little is known about the long-term effects of different leguminous species in aging tea plantations, particularly their roles in regulating carbon, nitrogen, and phosphorus cycling processes. Therefore, this study was conducted in a 40-year-old tea plantation of *Camellia sinensis* cv. “Fuding Dabai,” with intercropping of alfalfa (*Medicago sativa*, TAL), hairy vetch (*Vicia villosa,* THV), and Chinese milk vetch (*Astragalus sinicus,* TMV). The objectives were to systematically evaluate their effects on tea yield, soil physicochemical properties, enzyme activities (e.g., urease, sucrase, acid/alkaline phosphatase, amylase and catalase), and bacterial and fungal community structure, and to explore their potential roles in C–N–P cycling through functional predictions. This study aims to provide both theoretical insights and practical references for improving soil fertility and promoting sustainable management of aging tea plantation ecosystems.

## 2 Materials and methods

### 2.1 Study area

The field experiment was established in October 2022 at the Tea Plantation Base of the Jiangxi Academy of Cash Crops (28°22′N, 116°00′E), located in Jiangxi Province, China. The region has a subtropical monsoon climate, characterized by warm and humid conditions, abundant rainfall, and a long frost-free period. The experimental tea plants were 40-year-old *Camellia sinensis* cv. “Fuding Dabai,” planted with a row spacing of 1.5 m and a plant spacing of 20 cm, and were managed under light pruning.

The soil at the experimental site is derived from Quaternary red clay and classified as acidic red soil. Before the initiation of the experiment, the surface soil (0–20 cm) was analyzed and its properties were as follows: pH 4.48, organic matter 31.04 g·kg^−1^, total nitrogen 2.59 g·kg^−1^, total phosphorus 2.19 g·kg^−1^, total potassium 4.21 g·kg^−1^, alkali-hydrolyzed nitrogen 130.71 mg·kg^−1^, available phosphorus 115.34 mg·kg^−1^, and available potassium 158.25 mg·kg^−1^.

### 2.2 Experimental design and treatments

The experimental tea plants were *Camellia sinensis* cv. “Fuding Dabai” with an age of 40 years. Three leguminous green manure species were selected: alfalfa (*Medicago sativa*, TAL, cultivar Zhongmu No. 1), hairy vetch (*Vicia villosa*, THV, cultivar Xutie No. 1), and Chinese milk vetch (*Astragalus sinicus*, TMV, cultivar Yujiang Dayezi). A bare field without green manure was used as the control (CK). The experiment was conducted using a randomized block design (RBD), with three complete blocks established in total. Each block contained all four treatments, resulting in 12 plots (four treatments × three replicates), and each plot covered an area of 134 m^2^. Within each block, the plots were arranged consecutively, and concrete ridges were constructed between all plots to prevent water and soil exchange among treatments.

Green manures were sown in autumn and incorporated into the soil at full flowering in the following spring, with incorporation rates based on conventional seeding amounts. Detailed sowing rates, incorporation dates, and depths are provided in Table 1. The fertilizers used included rapeseed cake (organic matter content approximately 70%) and tea-specific compound fertilizer (N ≥ 18%, P₂O₅ ≥ 8%, K₂O ≥ 12%), with application dates, amounts, and depths also detailed in Table 1. Weed control was performed manually without the use of herbicides. Pest management included regular monitoring of tea plants and removal of infected leaves.

**Table 1:** Sowing practices, incorporation, and fertilization management of leguminous green manures.

Year	Sowing date	Type of green manure and seeding rate (kghm^−2^ per year)	Time of incorporation	Application of organic fertilizer	Topdressing with tea-specific fertilizer
2022	Oct. 30	Alfalfa (30), hairy vetch (30), and Chinese milk vetch (30)	–	Oct. 13 — Basal fertilizer 3,750 kg·hm^−2^, 30 cm depth	–
2023	Oct. 30		Mar. 21	Oct. 11 — Basal fertilizer 3,750 kg·hm^−2^, 30 cm depth	Feb. 23 — Fertilizer application: 600 kg·hm^−2^
2024			Mar. 26	–	Feb. 25 — Fertilizer application: 600 kg·hm^−2^

“–” indicates no treatment.

### 2.3 Analysis of soil physicochemical properties and enzyme activities

During the spring tea harvesting period on April 2, 2024, soil samples were randomly collected from the 0–20 cm layer in each plot using the five-point composite sampling method. This sampling represents early-season soil status only, and seasonal variations were not captured. After removing coarse roots and stones, the samples were brought back to the laboratory, air-dried, and passed through a 2 mm sieve for subsequent analysis. Each plot contained three replicates, and within each replicate, soil samples were collected from five points and composited to form a single sample for analysis. The air-dried samples were used for the determination of soil nutrient contents. Soil physicochemical properties were analyzed following the methods described in *Soil Agrochemical Analysis* (Bao, 2000). The measured indices included: soil pH (1:2.5 soil-to-water ratio, electrode method), organic matter (potassium dichromate oxidation–external heating method), total nitrogen (Kjeldahl method), total phosphorus (molybdenum–antimony colorimetric method after acid digestion), total potassium (flame photometry after acid digestion), alkali-hydrolyzed nitrogen (alkali-hydrolysis diffusion method), available phosphorus (Olsen method), and available potassium (NH_4_OAc extraction–flame photometry).

Fresh soil (0–20 cm) was collected using the five-point composite method from each replicated plot (n = 3). Samples were immediately placed in sterile bags, transported on ice, and stored at 4 °C before enzyme analyses. Amylase activity was measured using the 3,5-dinitrosalicylic acid (DNS) colorimetric method; catalase activity was determined by potassium permanganate titration; urease activity was measured using the phenol–sodium hypochlorite colorimetric method; sucrase activity was determined using the DNS colorimetric method; Acid phosphatase activity was measured using the p-nitrophenyl phosphate (pNPP) method due to its suitability and established use for soil samples. Alkaline phosphatase activity was analyzed using a fluorometric microplate assay to achieve higher sensitivity, as alkaline phosphatase activity in soil is generally lower and may not be accurately quantified by the pNPP colorimetric method. Tea yield was assessed by manually harvesting all one-bud–one-leaf shoots within a fixed area (1 m^2^) in each plot at each harvest period. Fresh weights were recorded and converted to yields per hectare based on planting density.

### 2.4 Microbial community sequencing and functional prediction

Soil samples were immediately stored at low temperature after collection, and total DNA was extracted using the FastDNA Spin Kit (MP Biomedicals, USA). The concentration and purity of the extracted DNA were determined using a NanoDrop 2000 spectrophotometer (Thermo Scientific, USA). For bacterial communities, the V3–V4 region of the 16S rRNA gene was amplified with primers 338F/806R, while for fungal communities, the ITS1 region was amplified with primers ITS1F/ITS2R. Amplification products were verified by agarose gel electrophoresis, purified, and then subjected to paired-end sequencing (2 × 300 bp) on the Illumina MiSeq platform.

Raw sequencing data were quality-filtered using fastp (v0.18.0) to remove reads containing ≥10% N bases, reads with ≥50% bases having Phred scores ≤20, and adapter-contaminated reads (Chen et al., 2018). Clean paired-end reads were then merged into tags using FLASH (v1.2.11) with a minimum overlap of 10 bp and a maximum mismatch rate of 2% (Magoč and Salzberg, 2011). the minimum sequencing depth for 16S amplicon data was 37,500 tags, and the minimum depth for ITS data was 70,000 tags. Subsequent quality trimming followed the strategy of Bokulich et al. (2013), in which tags were truncated at the first low-quality base (Q ≤ 3) within a 3-bp sliding window, and sequences retaining <75% of their original length were discarded. Operational taxonomic units (OTUs) were clustered at 97% similarity using the UPARSE algorithm implemented in USEARCH (v11.0.667) (Edgar, 2010), and chimeric sequences were removed using UCHIME (Edgar et al., 2011). Although ASV-based analysis provides higher taxonomic resolution, OTU clustering was retained to maintain consistency with previous research on tea plantation soils and to facilitate comparison with historical datasets. Representative sequences were taxonomically annotated against the SILVA (v138.2) and UNITE (v10.0) databases using the RDP naïve Bayesian classifier (Pruesse et al., 2007; Nilsson et al., 2019; Wang et al., 2007). Alpha-diversity indices, including Chao1, ACE, Shannon, Simpson, Pielou’s evenness, Good’s coverage, and PD-whole-tree, were calculated in R based on reference formulas from the mothur online repository, and rarefaction as well as rank-abundance curves were generated using ggplot2 (Wickham, 2011). Beta-diversity analyses were performed by computing weighted and unweighted UniFrac distance matrices using GUniFrac (Lozupone and Knight, 2005), while Jaccard and Bray–Curtis distance matrices were calculated from OTU abundance tables using vegan (Oksanen et al., 2010). Principal coordinates analysis (PCoA) was subsequently conducted based on these distance matrices using the vegan package.

Non-metric multidimensional scaling (NMDS), UPGMA clustering, and principal component analysis (PCA) were conducted using the vegan package in R (Oksanen et al., 2010). Community compositional differences were further evaluated using PERMANOVA (Adonis) and ANOSIM. For bacterial functional prediction, KEGG metabolic pathways were inferred using PICRUSt2 (version 2.5.3) (Langille et al., 2013), microbial phenotypes were categorized using BugBase (Ward et al., 2017), and ecological functional groups were assigned using FAPROTAX (version 1.2.10). For fungal communities, MetaCyc pathway predictions were generated using PICRUSt2 (Langille et al., 2013), and functional guilds were annotated using FUNGuild (Nguyen et al., 2016). It should be noted that PICRUSt2 predicts functions based on known reference genomes, assuming that phylogenetically related organisms share similar gene contents; thus, predictions may be limited by reference database completeness and taxonomic resolution. PICRUSt2 was applied to infer the potential metabolic functions of bacterial communities in carbon, nitrogen, and phosphorus pathways, while FUNGuild was used to classify fungal communities into ecological guilds. For FUNGuild, only assignments with confidence levels of “probable” or “highly probable” were considered to reduce misclassification. Correlation analyses were carried out using R software (version 4.2.1), with Spearman correlation and redundancy analysis (RDA) applied to explore the relationships between microbial communities and soil nutrient parameters.

### 2.5 Data analysis

All experimental data were organized using Excel 2019 and statistically analyzed with SPSS 26.0 software (QIIME2, RRID: RRID:SCR_021258). Prior to ANOVA, the normality of data distributions was assessed using the Shapiro–Wilk test, and homogeneity of variances was evaluated with Levene’s test. Differences among treatments for each variable were tested by one-way analysis of variance (ANOVA), with significance determined at *p <* 0.05. Multiple comparisons of means were performed using Duncan’s multiple range test. Correlation analyses were conducted using the Spearman method. Functional predictions of microbial communities were performed using PICRUSt2 (RRID:SCR_021127) and FUNGuild. Figures were generated with Origin 2021 (RRID:SCR_014212), R software (RRID:SCR_001905), and Adobe Illustrator (RRID:SCR_010279).

## 3 Results

### 3.1 Effects of intercropping tea plants with leguminous green manures on the tea plantation ecosystem

#### 3.1.1 Tea yield and agronomic traits

As shown in Table 2, the yield increase across the three harvesting seasons was primarily attributed to the consistent enhancement of bud density and the weight per 100 buds under the green-manure treatments. In the spring, summer, and autumn seasons, the THV treatment exhibited significantly higher bud density and bud weight compared with CK, with bud density increasing by 7.82–26.42% and bud weight increasing by 9.51–16.20%. Correspondingly, the yield of THV remained significantly higher than that of CK throughout all seasons, indicating a stable and persistent yield-promoting effect.

**Table 2:** Tea yield and agronomic traits under different leguminous green manure intercropping treatments.

Season	Treatment	Bud density (buds·m^−2^)	Weight per 100 buds (g)	Yield (kg·hm^−2^)
Spring tea	CK	918.00 ± 15.59b	27.93 ± 0.70b	601.00 ± 51.42b
	TAL	1134.00 ± 47.62a	32.03 ± 0.93a	899.17 ± 97.16a
	THV	1044.00 ± 54.99a	30.93 ± 2.58a	843.00 ± 17.52a
	TMV	1023.00 ± 13.75a	30.53 ± 1.10a	839.50 ± 30.13a
Summer tea	CK	825.00 ± 27.50b	25.50 ± 0.72b	532.90 ± 32.26b
	TAL	1032.00 ± 93.67a	29.63 ± 0.50a	750.77 ± 46.57a
	THV	1043.00 ± 59.92a	29.63 ± 1.01a	756.83 ± 97.58a
	TMV	1005.00 ± 88.79a	27.67 ± 0.35ab	603.63 ± 73.45b
Autumn tea	CK	639.00 ± 9.00b	24.93 ± 1.42b	429.77 ± 25.39c
	TAL	651.00 ± 68.15b	26.60 ± 0.50a	616.23 ± 50.67ab
	THV	689.00 ± 32.45a	27.30 ± 0.53a	650.17 ± 63.88a
	TMV	659.00 ± 42.53b	27.50 ± 0.53a	619.17 ± 51.06ab

CK, clean tillage; TAL, tea intercropped with alfalfa; THV, tea intercropped with hairy vetch; TMV, tea intercropped with Chinese milk vetch. Data are presented as mean ± SE of three replicates. Different letters within the same column indicate significant differences at the 5% level according to LSD test (the same applies hereafter).

Overall, both TAL and THV demonstrated pronounced yield advantages, with yields exceeding those of CK by 40.88–49.61% and 40.27–51.28%, respectively, across the three seasons. The incorporation of green manures generally enhanced bud emergence and growth vigor of tea plants, among which THV consistently produced the most stable and substantial yield improvements, whereas the effect of TMV was comparatively moderate.

#### 3.1.2 Effects of intercropping tea plants with leguminous green manures on soil properties

As shown in Table 3, the green manure treatments resulted in measurable differences in soil physicochemical properties. pH and soil organic matter (SOM): Soil pH was lowest in the CK treatment. The THV treatment had a pH of 4.67, which was 5.42% higher than the CK(*p <* 0.05, LSD test). All leguminous green manure treatments increased SOM compared with CK, with values 29.02–32.26% higher (*p <* 0.05, LSD test). Nitrogen and phosphorus: All green manure treatments increased soil total nitrogen by 16.59–23.96% relative to CK (*p <* 0.05, LSD test). Soil total phosphorus under THV and TMV was 3.12 g·kg^−1^ and 3.53 g·kg^−1^, respectively, which were 47.17 and 66.51% higher than CK (*p <* 0.05, LSD test). Available phosphorus (AP) was highest in the THV treatment, showing a 112.12% increase compared with CK (*p <* 0.05, LSD test). Potassium: Under the THV treatment, soluble potassium content was 19.88–31.29% higher than in the other treatments, and total potassium was 56.97% higher than in CK (*p <* 0.05, LSD test).

**Table 3:** Effects of intercropping tea plants with green manures on soil properties.

Treatment	pH g·kg^−1^	SOM g·kg^−1^	TN g·kg^−1^	TP g·kg^−1^	TK g·kg^−1^	AN mg·kg^−1^	AP mg·kg^−1^	AK mg·kg^−1^
CK	4.43 ± 0.05b	28.05 ± 2.06b	2.17 ± 0.17b	2.12 ± 0.14b	4.02 ± 0.82b	144.03 ± 12.18b	99.23 ± 3.25c	147.87 ± 0.30b
TAL	4.53 ± 0.08ab	36.19 ± 0.22a	2.53 ± 0.21a	2.33 ± 0.29b	5.02 ± 0.26ab	185.63 ± 7.30a	179.31 ± 2.37b	167.30 ± 4.45b
THV	4.67 ± 0.12a	37.10 ± 0.96a	2.69 ± 0.07a	3.12 ± 0.40a	6.31 ± 1.21a	180.58 ± 7.29a	210.69 ± 6.02a	193.27 ± 20.71a
TMV	4.60 ± 0.17ab	36.59 ± 1.08a	2.63 ± 0.22a	3.53 ± 0.20a	5.32 ± 0.57ab	187.20 ± 9.56a	170.73 ± 17.95b	161.04 ± 11.76b

CK, clean tillage; TAL, tea intercropped with alfalfa; THV, tea intercropped with hairy vetch; TMV, tea intercropped with Chinese milk vetch. SOM, soil organic matter; TN, total nitrogen; TP, total phosphorus; TK, total potassium; AN, alkali-hydrolyzable nitrogen; AP, available phosphorus; AK, available potassium. Data are presented as mean ± SE of three replicates. Different letters within the same column indicate significant differences at the 5% level according to LSD test (the same applies hereafter).

#### 3.1.3 Soil enzyme activities

As shown in Table 4, different leguminous green manure treatments had significant effects on soil enzyme activities in tea plantations. Compared with CK, TAL, THV, and TMV significantly increased amylase activity by 53.05, 39.63, and 78.05%, respectively (*p <* 0.05). Catalase activity under the THV treatment was significantly higher than in all treatments except TAL, exceeding the other treatments by 29.60–47.27% (*p <* 0.05). Both urease and sucrase activities were significantly enhanced under leguminous green manure treatments compared with CK, with increases of 91.78–101.11% and 61.72–63.18%, respectively (*p <* 0.05). The acid phosphatase activity under the THV and TMV treatments was significantly higher than that under TAL, by 19.66 and 15.94%, respectively (*p <* 0.05). The alkaline phosphatase activity in CK was significantly lower than in the other treatments, by 25.52 and34.67% (*p <* 0.05).

**Table 4:** Soil enzyme activities under different leguminous green manure intercropping treatments in tea plantations.

Treatment	Amylase mg·(g·d)^−1^	Catalase (U·g^−1^)	Urease mg·(g·h)^−1^	Sucrase (U·g^−1^)	Acid phosphatase (U·g^−1^)	Alkaline phosphatase (U·g^−1^)
CK	1.64 ± 0.13b	2.50 ± 0.18c	427.04 ± 98.25b	4.78 ± 0.50b	53706.46 ± 3032.75ab	5575.79 ± 686.30b
TAL	2.51 ± 0.28a	2.87 ± 0.19ab	819.06 ± 53.86a	7.80 ± 0.52a	48757.88 ± 5959.92b	7508.94 ± 190.68a
THV	2.29 ± 0.43a	3.24 ± 0.07a	858.85 ± 49.34a	7.73 ± 0.67b	58341.26 ± 3152.41a	7231.22 ± 470.17a
TMV	2.92 ± 0.63a	2.20 ± 1.20c	821.58 ± 71.42a	7.75 ± 0.43a	56529.85 ± 1835.17a	6998.15 ± 534.49a

CK, clean tillage; TAL, tea intercropped with alfalfa; THV, tea intercropped with hairy vetch; TMV, tea intercropped with Chinese milk vetch. Data are presented as mean ± SE of three replicates. Different letters within the same column indicate significant differences at the 5% level according to LSD test (the same applies hereafter).

### 3.2 Effects of intercropping tea plants with leguminous green manures on soil microbial communities

#### 3.2.1 Bacterial and fungal community composition

**Figure 1:**
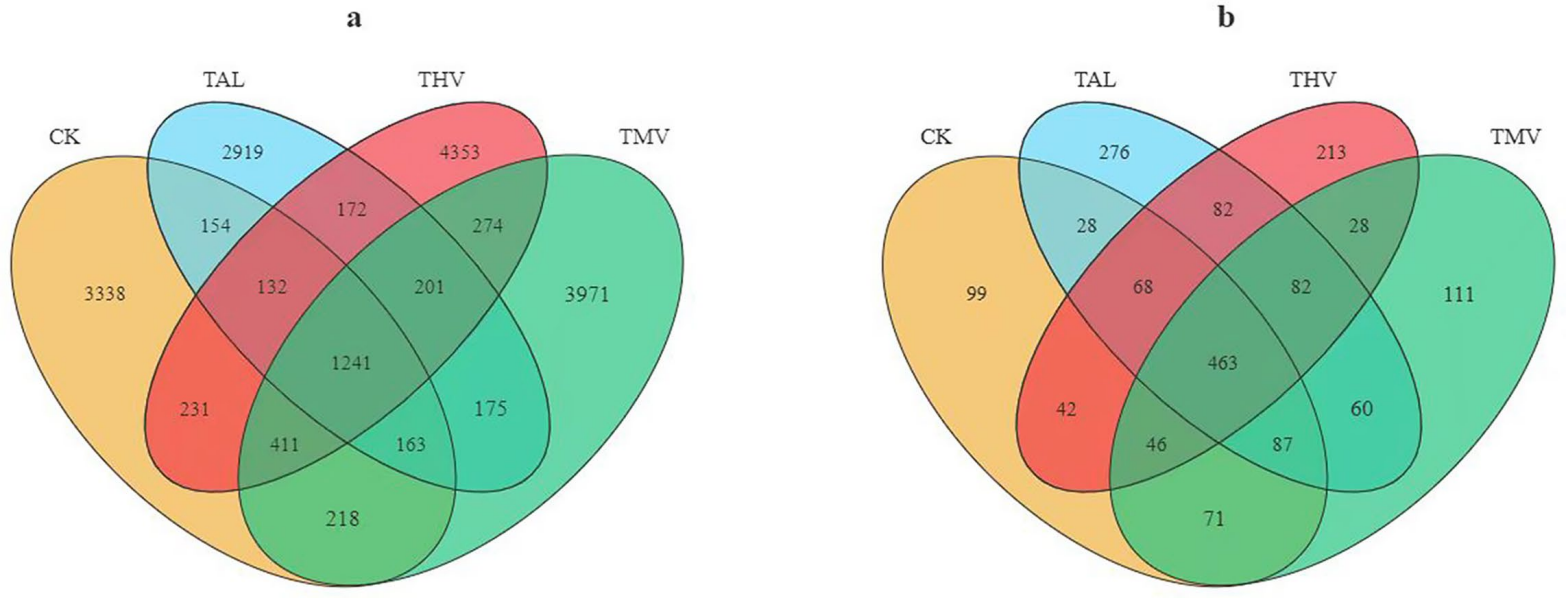
Venn diagrams showing the distribution of bacterial **(a)** and fungal **(b)** OTUs under different leguminous green manure treatments. Note: CK, clean tillage; TAL, tea intercropped with alfalfa; THV, tea intercropped with hairy vetch; TMV, tea intercropped with Chinese milk vetch. The same applies hereafter.

As shown in Figure 1a, in the bacterial Venn diagram, the numbers of OTUs in tea plantation soils were CK (4198), TAL (5157), THV (7015), and TMV (6654). A total of 1,241 OTUs were shared, accounting for 29.56, 24.06, 17.69, and 18.65% of CK, TAL, THV, and TMV, respectively. The numbers of unique OTUs in TAL, THV, and TMV were all higher than in CK, with increases of 22.84, 67.10, and 58.50%, respectively (*p <* 0.05). Among them, THV had the highest number of unique OTUs, 5.43–67.10% higher than the other treatments (*p <* 0.05). In the fungal Venn diagram (Figure 1b), the numbers of OTUs were CK (904), TAL (1146), THV (1024), and TMV (948). A total of 463 OTUs were shared, representing 51.22, 40.40, 45.21, and 48.84% of CK (*p <* 0.05), TAL, THV, and TMV, respectively. The numbers of unique OTUs under TAL and THV were 11.91–26.77% and 8.02–13.27% higher than those in the other treatments, respectively (*p <* 0.05). The numbers of unique bacterial OTUs under leguminous green manure treatments were higher than CK, with THV showing the highest number of unique OTUs (*p <* 0.05).

**Figure 2:**
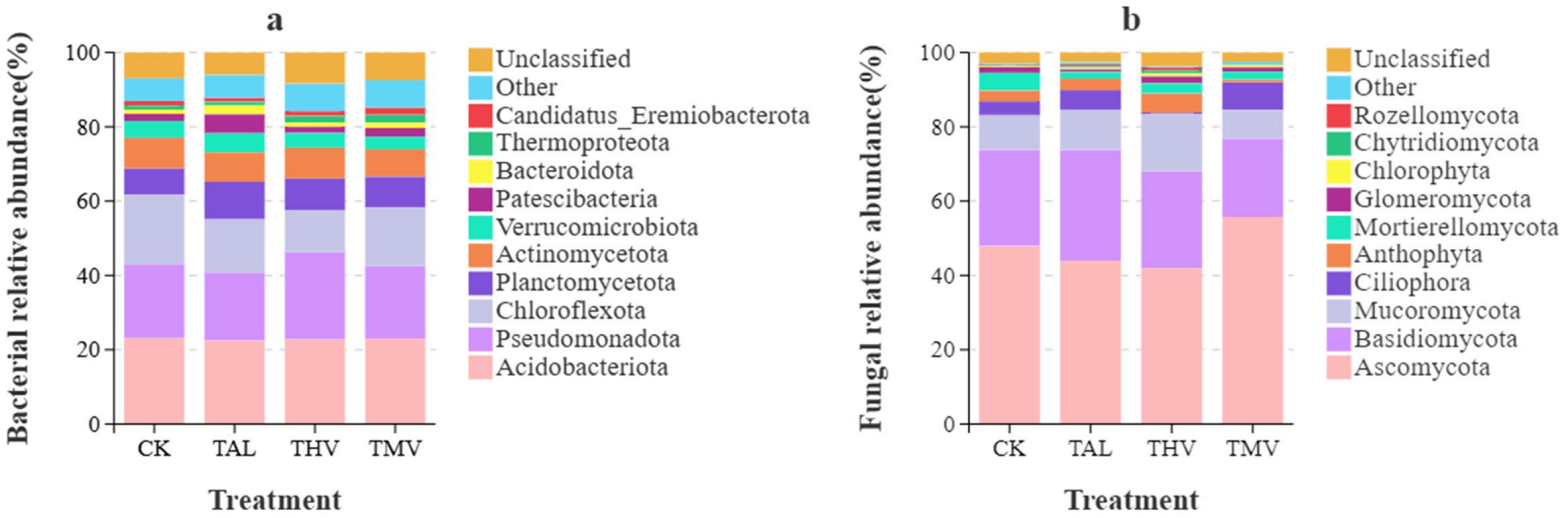
Relative abundance of bacterial **(a)** and fungal **(b)** communities under different leguminous green manure treatments.

As shown in Figure 2a, at the phylum level, the top five dominant bacterial groups in tea plantation soils were *Acidobacteriota*, *Pseudomonadota*, *Chloroflexota*, *Planctomycetota*, and *Actinomycetota*, together accounting for 73.12–77.04% of the total sequences (*p* < 0.05). Although the dominant phyla were generally consistent across treatments, their relative abundances varied. Under leguminous green manure treatments (TAL, THV, TMV), the relative abundances of *Pseudomonadota* and *Planctomycetota* increased, while *Acidobacteriota* and *Chloroflexota* were more abundant in the CK treatment. Notably, Pseudomonadota under THV accounted for the highest proportion among all treatments, 18.23–20.83% higher than the others, whereas *Planctomycetota* under TAL reached the highest proportion, 20.38–45.56% greater than the other treatments (*p <* 0.05).

As shown in Figure 2b, the fungal community at the phylum level was mainly composed of *Ascomycota* and *Basidiomycota*, with a combined relative abundance exceeding 70%. *Ascomycota* dominated in all treatments and was particularly enriched under TMV, where its relative abundance was 16.38–32.75% higher than in the other treatments (*p <* 0.05). In contrast, Basidiomycota was more abundant under TAL, with relative abundances 14.85–41.87% higher than in the other treatments (*p <* 0.05). Meanwhile, the proportions of *Mucoromycota* and *Mortierellomycota* also increased under leguminous green manure treatments, suggesting their involvement in organic matter decomposition and nutrient transformation. In contrast, the fungal community composition under CK was relatively simple, indicating that the absence of green manure inputs reduced fungal diversity and ecological functional potential. Overall, intercropping tea plants with leguminous green manures was associated with changes in the relative abundance of bacterial and fungal communities. In bacterial communities, the relative abundances of groups such as *Pseudomonadota* and *Chloroflexota* were higher under green manure treatments, while *Ascomycota* and *Basidiomycota* remained the dominant fungal phyla across treatments. These observations reflect treatment-related differences in soil microbial community composition.

#### 3.2.2 Diversity of soil bacterial and fungal communities

##### 3.2.2.1 Alpha diversity

**Figure 3:**
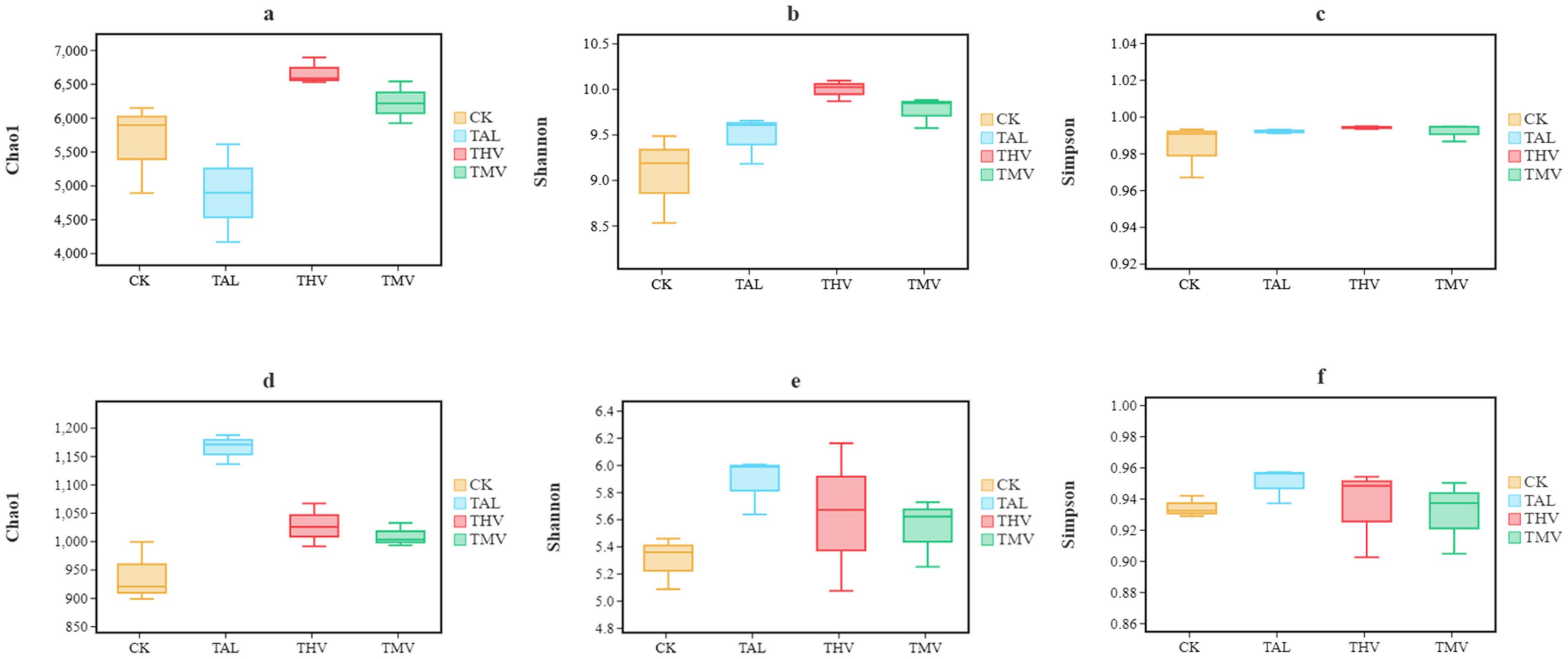
Alpha diversity indices of bacterial **(a–c)** and fungal **(d–f)** communities at the species level under different leguminous green manure treatments.

Within each group, the bacterial communities under the four treatments were evaluated. As shown in Figure 3, compared with the control (CK), THV and TMV significantly increased bacterial community richness (Chao1 index, *p <* 0.05, *t*-test) and diversity (Shannon index, *p <* 0.05, t-test), with the greatest increase observed under THV. However, community evenness (Simpson index, *p* > 0.05, *t*-test) showed no significant change. For fungal communities, the evaluation of the four treatments revealed that leguminous green manure treatments (TAL, THV, TMV) significantly increased community richness (Chao1 index, *P* = < 0.05, *t*-test), while no significant differences were observed in community diversity (Shannon index, *p* > 0.05, *t*-test) or evenness (Simpson index, *p* > 0.05, *t*-test). In summary, leguminous green manures significantly enhanced the richness and diversity of bacterial communities, whereas fungal communities only exhibited an increase in richness, with hairy vetch (THV) showing the strongest effect.

##### 3.2.2.2 Beta diversity

**Figure 4:**
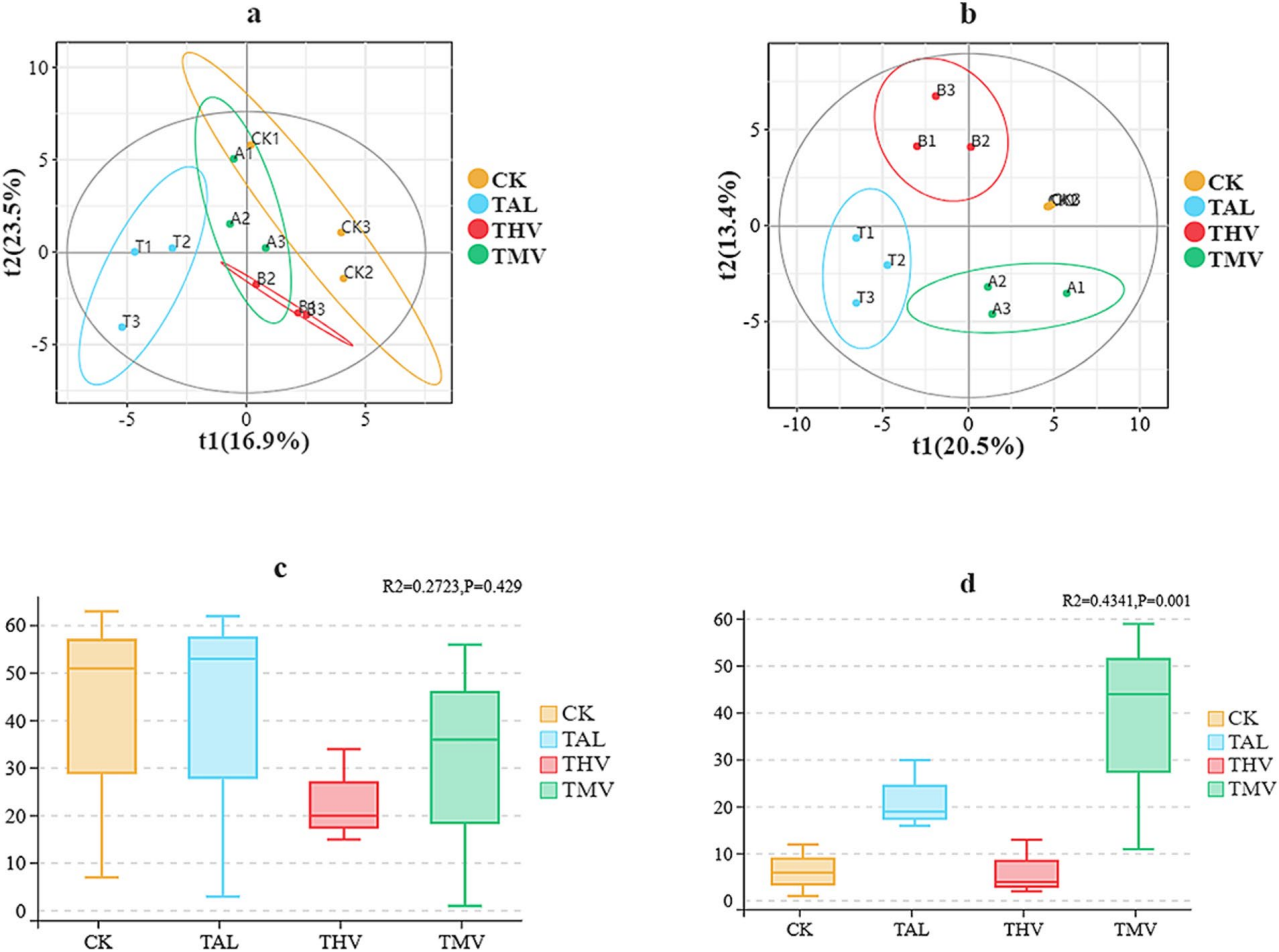
Principal coordinate analysis (PCoA) of bacterial **(a)** and fungal **(b)** community structures under different leguminous green manure treatments, with corresponding PERMANOVA (Adonis) results for bacteria **(c)** and fungi **(d)**.

As shown in Figure 4, Principal coordinate analysis (PCoA) based on Bray–Curtis distances revealed differences in community composition among treatments for both bacterial and fungal communities (Figures 4a,b). To statistically evaluate these patterns, permutational multivariate analysis of variance (PERMANOVA, Adonis) was performed. For bacterial communities, the first two principal components explained 40.4% of the variation. CK samples were clustered in the positive direction of PC1 and PC2, clearly separated from the intercropping treatments. TAL and TMV were clustered together in the negative direction of PC1 and PC2, indicating a high degree of similarity, whereas THV was positioned toward the positive direction of PC1, maintaining distinctiveness. For fungal communities, the first two principal components explained 33.9% of the variation. CK samples were still located in the positive direction but were more dispersed, suggesting greater internal heterogeneity. TAL and TMV were clustered together in the negative direction, while THV was positioned toward the negative direction of PC1 and the positive direction of PC2, showing distinct differentiation. For bacterial communities, PERMANOVA results indicated that treatment explained 27.23% of the total variation; however, this effect was not statistically significant (R^2^ = 0.2723, *p* = 0.429; Figure 4c). In contrast, fungal community composition differed significantly among treatments, with PERMANOVA showing that treatment accounted for 43.41% of the variation (R^2^ = 0.4341, *p* = 0.001; Figure 4d), indicating a strong treatment effect on fungal beta diversity. In summary, CK and leguminous green manure intercropping treatments differed significantly in soil microbial community structure. CK exhibited the most pronounced separation, TAL and TMV displayed similar community structures, while THV showed stronger independence and distinctiveness.

### 3.3 Functional analysis of microbial communities

**Figure 5:**
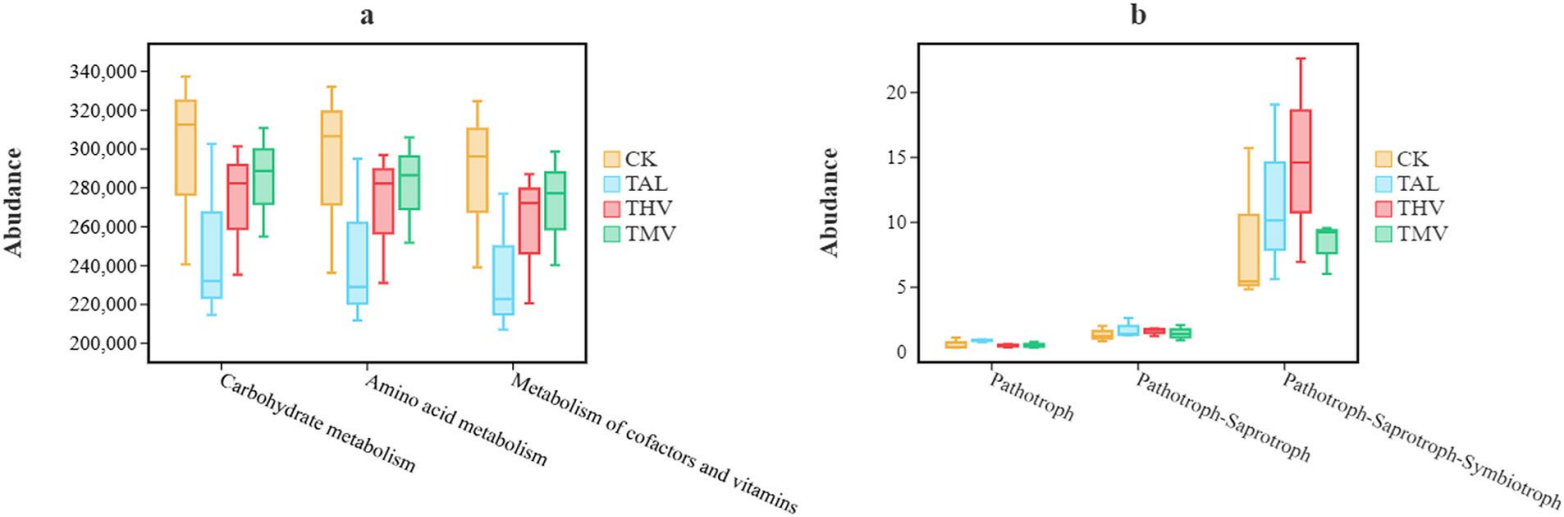
Predicted functional groups of bacterial **(a)** and fungal **(b)** communities under different leguminous green manure treatments.

For bacterial metabolic functions predicted by PICRUSt2 (Figure 5a), the CK group showed higher relative abundances in several pathways. Specifically, the predicted abundances of carbohydrate metabolism, amino acid metabolism, and cofactor and vitamin metabolism pathways were 8.49–11.43%, 8.52–12.54%, and 8.71–17.30% higher than those in the other treatments, respectively (*p <* 0.05). For fungal functional guilds (Figure 5b), the abundance of pathotrophs remained consistently low with no significant differences among treatments. In contrast, the abundance of pathotroph–saprotroph–symbiotroph guilds was markedly higher, with significant differences across treatments. Among them, THV showed the highest abundance, which was 23.27–43.09% higher than in the other treatments (*p <* 0.05). In summary, intercropping tea with leguminous green manures modulated bacterial metabolic pathways and fungal functional guilds, thereby enhancing microbial contributions to organic matter decomposition and nutrient cycling. For fungal communities, functional guild composition showed relatively conservative responses compared with bacterial functional profiles. Although significant differences were detected in mixed guilds (pathotroph–saprotroph–symbiotroph), the overall functional structure of fungi remained stable across treatments, indicating a lower short-term sensitivity of fungal functional traits to green manure intercropping.

It should be noted that the functional profiles shown in Figure 5 are based on predictive approaches rather than direct measurements of functional gene abundance or activity. Specifically, PICRUSt2 and FUNGuild infer microbial functional potential from taxonomic information, assuming phylogenetic conservation of functional traits. As a result, these predictions may not fully capture actual *in situ* metabolic activity, particularly for fungal communities, where functional annotation is constrained by reference database coverage and taxonomic resolution. Therefore, the functional differences observed in Figure 5 should be interpreted as indicative of potential functional shifts rather than quantitative evidence of realized biogeochemical processes.

### 3.4 Correlation analysis between soil environmental factors and bacterial and fungal community structures

**Figure 6:**
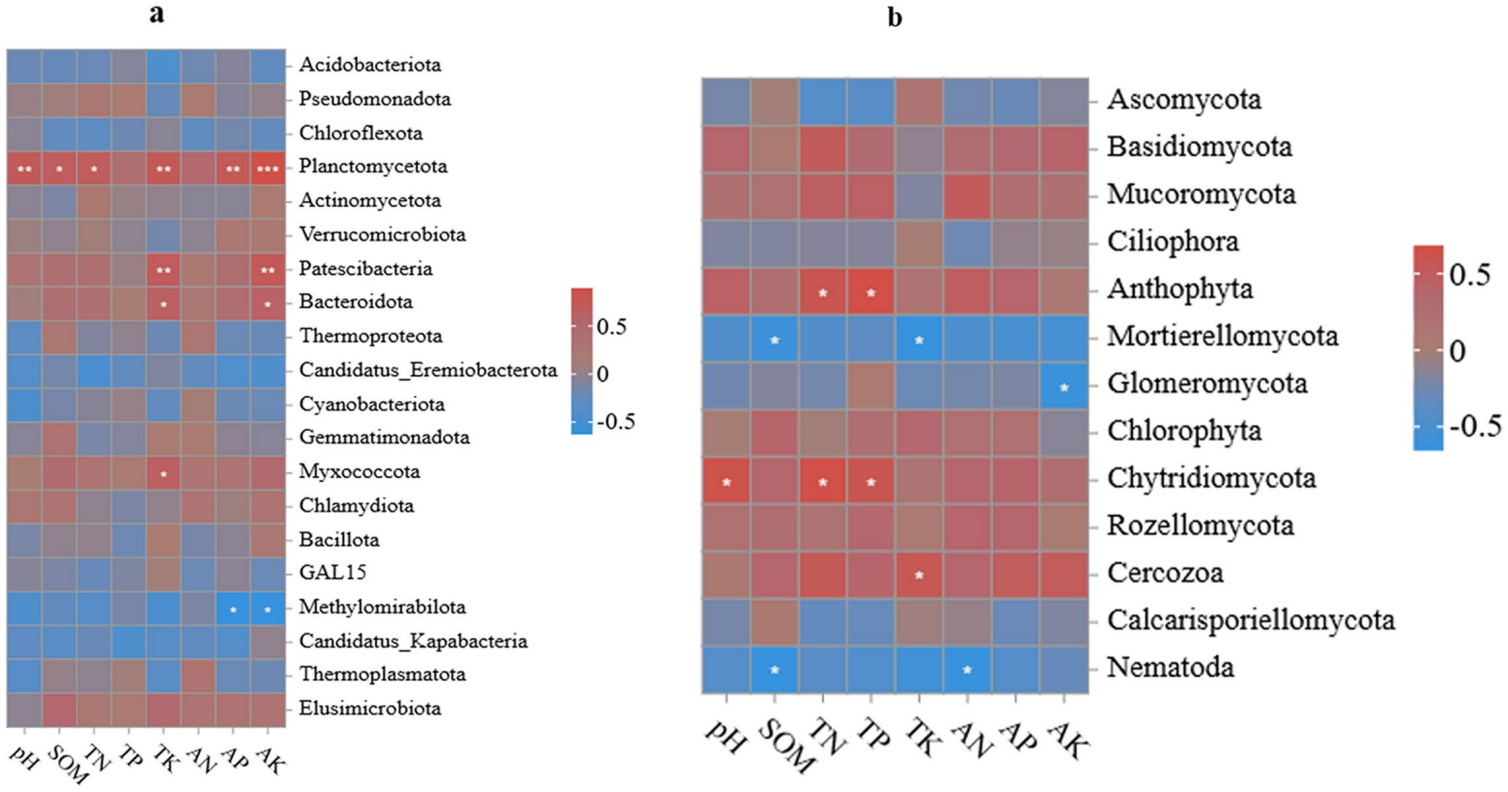
Cluster heatmaps showing the relationships between bacterial **(a)** and fungal **(b)** communities and soil chemical properties under different leguminous green manure treatments. Note: SOM, soil organic matter; TN, total nitrogen; TP, total phosphorus; TK, total potassium; AN, alkali-hydrolyzable nitrogen; AP, available phosphorus; AK, available potassium.

Based on the correlation analysis shown in Figure 6, intercropping green manure markedly altered the response patterns between dominant microbial taxa and soil physicochemical properties in the tea garden. Within the bacterial community, most major taxa showed positive associations with soil fertility indicators. *Planctomycetota* exhibited strong positive correlations with pH, soil organic matter, total nitrogen, total potassium, and available phosphorus and potassium, with particularly strong correlations with pH, total potassium, available phosphorus, and available potassium (*p <* 0.01), and significant correlations with organic matter and total nitrogen (*p <* 0.05). *Patescibacteria* and *Bacteroidota* showed notable positive correlations with potassium-related variables (total potassium and available potassium;(*p <* 0.01) for *Patescibacteria*, *p <* 0.05 for Bacteroidota). In contrast, *Methylomirabilota* was negatively correlated with available phosphorus and available potassium (*p <* 0.05), indicating divergent ecological responses of different taxa to the same nutrient gradients.

Compared with bacteria, fungal taxa responded more strongly to soil organic matter and phosphorus availability. *Mortierellomycota* showed negative correlations with organic matter and total potassium (*p <* 0.05), whereas *Chlorophyta* was positively correlated with pH, total phosphorus, and total potassium (*p <* 0.01). *Cercozoa* was positively correlated with total potassium (*p <* 0.05), while some taxa (e.g., *Nematoda*) displayed negative correlations with organic matter and alkali-hydrolyzable nitrogen (*p <* 0.05). Overall, nutrient and pH shifts induced by green manure intercropping caused dominant bacterial taxa to be primarily associated with readily available nutrients and soil acidity–alkalinity, whereas dominant fungal taxa were more strongly shaped by organic matter and phosphorus levels. This correlation pattern indicates that green manure not only improves soil conditions but also reshapes how microbial groups respond to key soil variables.

## 4 Discussion

### 4.1 Tea yield and soil nutrient improvement

This study showed that leguminous green manure intercropping was associated with increased tea yield, Among the treatments, THV maintained comparatively higher production across seasons, with the highest value observed in autymn (650.17 kg·hm^−2^). Higher yields under THV coincided with increases in soil organic matter and nutrient availability, consistent with previous findings. Possible mechanisms reported in earlier studies include biological nitrogen inputs and residue-derived organic matter, which may support nutrient turnover (Xiang et al., 2021; Ma et al., 2017). In addition, the increase in organic matter improved soil buffering capacity, mitigated acidification, and optimized the rhizosphere environment, which facilitated nutrient uptake and bud growth (Huang et al., 2022; Bai et al., 2022). Similar trends have been observed in other cropping systems, where legume integration corresponded with improved soil conditions (Gao et al., 2023; Xie et al., 2024). In tea plantations, Chen et al. (2025) reported that intercropping legumes improved soil properties and increased tea yield by regulating rhizosphere microbial communities; Zhou et al. (2024) found that green manures significantly enhanced soil enzyme activities and bacterial diversity; while Duan et al. (2024) highlighted their role in improving key tea quality components and yield.

Overall, leguminous green manure intercropping improved soil conditions and supported tea yield performance, with THV showing the greatest response under the conditions of this study.

### 4.2 Improvement of soil physicochemical properties and its ecological significance

This study highlights the key role of leguminous green manure intercropping in restoring soil ecological processes in aging tea plantations, beyond merely improving soil nutrients. Long-term monoculture often causes soil acidification, structural degradation, and weakened microbial functions, reducing rhizosphere activity and plant resilience (Yan et al., 2020). Our results show that green manure restores degraded soil functions by altering carbon and nitrogen inputs and rhizosphere processes. Leguminous green manure enhances microbial C–N coupling via nitrogen fixation and increased rhizosphere carbon, improving nitrogen mineralization and organic matter turnover (Cong et al., 2022). It also stimulates microbial growth and community diversity, boosting system functional potential (Wang S. et al., 2023). By improving soil pH, green manure enhances enzyme activity, stabilizes microbial networks, and increases soil resilience (Wagg et al., 2012). Furthermore, it increases phosphorus and potassium availability, reflecting enhanced microbial nutrient acquisition and higher soil utilization efficiency (Ma et al., 2023). In summary, leguminous green manure intercropping improves soil fertility, restores microbial functions, and enhances nutrient bioavailability, reconstructing soil ecological processes and supporting long-term tea plantation productivity (Cong et al., 2022; Wang T. et al., 2023).

Similar effects have also been reported in other cropping systems, such as rice and wheat, where green manures increased soil organic matter, improved nutrient availability, and raised soil pH (Liang et al., 2023; Ma et al., 2023). This study confirms that these mechanisms are equally applicable to perennial tea systems, particularly in aging plantations, where leguminous green manures play a significant role in restoring soil fertility. Overall, the legume treatments improved soil properties under the conditions of this study, with THV presenting the highest level of soil improvement among treatments.

### 4.3 Changes in soil enzyme activities and nutrient cycling

This study demonstrated that intercropping tea with leguminous green manures significantly enhanced the activities of multiple key soil enzymes involved in carbon (C), nitrogen (N), and phosphorus (P) cycling, with different treatments showing distinct functional features. Different treatments exhibited distinct enzyme response patterns; for example, elevated urease and sucrase activities under TAL aligned with previous observations (Wang et al., 2022). TMV showed higher amylase and acid phosphatase activities, consistent with studies relating these enzymes to substrate decomposition (Liu et al., 2024). Increased catalase activity under THV agrees with reports suggesting a role in oxidative regulation in rhizosphere environments (Yang et al., 2024). Green manure treatments were associated with higher activities of urease, sucrase, acid phosphatase, and catalase, which are enzymes involved in nitrogen transformation, carbon availability, and organic phosphorus mobilization as reported in previous studies (Kucerik et al., 2024). These patterns suggest potential shifts in nutrient-related microbial processes, although direct biogeochemical rates were not measured in this study.

Similar mechanisms have been validated in other cropping systems. For example, in orchards and perennial systems, green manures have been widely reported to significantly enhance the activities of *β*-glucosidase, urease, and phosphatase, thereby accelerating nutrient cycling and improving soil nutrient availability (Kama et al., 2025; He et al., 2023). This suggests that the mechanism by which green manures stimulate soil enzyme activities to enhance nutrient cycling has broad applicability. In summary, leguminous green manure intercropping was associated with increased activities of enzymes linked to C, N, and P cycling, with THV showing the highest response among treatments.

### 4.4 Responses of microbial community structure and diversity

This study showed that intercropping tea with leguminous green manures was associated with changes in soil microbial community composition and diversity, with different treatments exhibiting distinct responses. For bacteria, THV resulted in higher OTU numbers and increased Chao1 and Shannon indices compared with the control (Wang et al., 2025a,b,c). For fungi, TAL showed a higher OTU count than the control, while Shannon and Simpson indices did not differ among treatments, indicating that fungal diversity remained relatively stable.

Beta-diversity analysis based on PLS-DA indicated clear separation between CK and the green manure treatments, with the greatest distinction observed under THV. Shifts in bacterial community composition included higher relative abundances of phyla associated with nitrogen cycling and organic matter turnover, such as *Pseudomonadota* and *Planctomycetota*, which is consistent with previous findings in perennial systems (Shao et al., 2024; Duan et al., 2024). In contrast, fungal communities showed limited variation across treatments, and dominant groups such as *Ascomycota* and *Basidiomycota* remained consistently abundant, aligning with reports of their stable roles in carbon decomposition processes (Huang et al., 2023a). Similar patterns have been documented in other tea-based and intercropping systems, where bacterial communities responded more strongly to management practices than fungi (Shen and Lin, 2021; Yang et al., 2024).

Overall, bacterial communities appeared more responsive to leguminous green manure intercropping than fungal communities under the conditions of this study, with THV showing the largest shift among treatments. This study was based on a single site and year, and functional interpretations relied on PICRUSt2 predictions without direct measurements of nutrient transformation, so the conclusions should be interpreted with caution. Future work should include multi-year and multi-region trials and incorporate approaches such as metagenomics or isotope tracing to verify microbial functions and ecological effects. Optimizing legume species and management strategies may further improve soil health and productivity in acidified tea plantations.

### 4.5 Functional predictions and ecological mechanisms

The functional predictions in this study, generated using PICRUSt2, indicated that leguminous green manure intercropping was associated with shifts in microbial functional potential related to nutrient-cycling pathways. For bacteria, higher predicted abundances of pathways associated with amino acid metabolism, carbohydrate metabolism, and energy metabolism were observed under legume treatments, with the greatest increase under THV (Walker et al., 2023). For fungi, the predicted functional profiles showed that dominant groups such as Ascomycota and Basidiomycota maintained high representation in pathways associated with organic carbon and phosphorus transformation, which was consistent with their relative abundances (Park et al., 2023; Carciochi et al., 2024). These functional patterns corresponded with the observed increases in enzyme activities involved in C, N, and P cycling; however, direct measurements of biogeochemical rates were not conducted in this study. Similar trends have been reported in other perennial and orchard systems, where green manure management was associated with changes in microbial functional potential and nutrient-related enzyme activities (Feng et al., 2023; Yang et al., 2024). Overall, leguminous green manure intercropping was associated with shifts in predicted microbial functions and enzyme activities under the conditions of this study, with THV showing the largest response among treatments.

Compared with bacteria, fungal functional guilds exhibited relatively limited responses to green manure intercropping. This pattern may be attributed to several factors. First, fungal communities in perennial tea plantation soils are often functionally conservative, with dominant saprotrophic and symbiotrophic guilds maintaining stable ecological roles despite changes in nutrient inputs, a phenomenon widely observed in perennial and woody agroecosystems (Bahram et al., 2018). Second, fungi generally respond to management practices over longer temporal scales than bacteria, particularly in woody perennial systems, where fungal-mediated decomposition and nutrient acquisition processes are closely linked to long-term organic matter accumulation and substrate quality rather than short-term nutrient inputs (Banerjee et al., 2019). Third, fungal functional annotation based on FUNGuild relies on taxonomic inference rather than direct functional gene quantification, which may constrain the detectable resolution of functional shifts and has been explicitly recognized as a methodological limitation in recent studies (Põlme et al., 2020). Therefore, the relatively modest changes observed in fungal functional guilds likely reflect ecological stability rather than a lack of biological relevance. It should be noted that fungal functional profiles were inferred using FUNGuild based on taxonomic information, and thus represent potential ecological roles rather than direct measurements of functional activity.

## 5 Conclusion

This study demonstrated that intercropping tea with leguminous green manures can effectively improve soil health and enhance microbial functional potential. Among the tested species, hairy vetch (THV) showed the greatest impact, indicating its high suitability for restoring soil ecological functions in aging tea plantations. These findings highlight the broader significance of integrating green manures into perennial cropping systems as a sustainable management practice to mitigate soil degradation and promote ecosystem resilience. Future research should focus on applying multi-omics approaches, including metagenomics, transcriptomics, and metabolomics, to uncover the molecular mechanisms underlying microbial responses to green manure. In addition, long-term, large-scale field experiments are needed to validate the ecological benefits and practical applicability of green manure intercropping in diverse tea plantation systems.

## Data Availability

The original contributions presented in the study are included in the article/supplementary material, further inquiries can be directed to the corresponding authors.
